# Postmortem Findings for 7 Neonates with Congenital Zika Virus Infection

**DOI:** 10.3201/eid2307.162019

**Published:** 2017-07

**Authors:** Anastácio Q. Sousa, Diane I.M. Cavalcante, Luciano M. Franco, Fernanda M.C. Araújo, Emília T. Sousa, José Telmo Valença-Junior, Dionne B. Rolim, Maria E.L. Melo, Pedro D.T. Sindeaux, Marialva T.F. Araújo, Richard D. Pearson, Mary E. Wilson, Margarida M.L. Pompeu

**Affiliations:** Federal University of Ceará, Fortaleza, Brazil (A.Q. Sousa, D.I.M. Cavalcante, L.M. Franco, E.T. Sousa, J.T. Valença-Junior, P.D.T. Sindeaux, M.M.L. Pompeu);; Serviço de Verificação de Óbitos-SVO, Fortaleza (L.M. Franco, E.T. Sousa, J.T. Valença-Junior);; Ceará State Central Public Health Laboratory, Fortaleza (F.M.C. Araújo, M.E.L. Melo);; University of Fortaleza, Fortaleza (D.B. Rolim); Ceará State Secretariat of Health, Fortaleza (D.B. Rolim);; Evandro Chagas Institute, Belém, Brazil (M.T.F. Araújo);; University of Virginia School of Medicine, Charlottesville, Virginia, USA (R.D. Pearson);; University of California, San Francisco, California, USA (M.E. Wilson);; Harvard T.H. Chan School of Public Health, Boston, Massachusetts, USA (M.E. Wilson)

**Keywords:** Zika virus, microcephaly, autopsy, postmortem, Brazil, viruses, congenital, neonates

## Abstract

Postmortem examination of 7 neonates with congenital Zika virus infection in Brazil revealed microcephaly, ventriculomegaly, dystrophic calcifications, and severe cortical neuronal depletion in all and arthrogryposis in 6. Other findings were leptomeningeal and brain parenchymal inflammation and pulmonary hypoplasia and lymphocytic infiltration in liver and lungs. Findings confirmed virus neurotropism and multiple organ infection.

From the discovery of Zika virus in Uganda in 1947 through 2007, when an outbreak occurred on Yap Island, Micronesia, only sporadic cases of human infection had been reported (*1*). In early 2015, the virus emerged in Brazil (*2*). Its role in a major public health crisis became apparent when links between Zika virus infection and microcephaly and Guillain-Barré syndrome were established (*1*). Cases of microcephaly associated with Zika virus have been well documented (*3*–*7*). We report a case series of postmortem findings that strengthen this association.

## The Study

Postmortem examinations were performed on 7 neonates from the state of Ceará, in northeastern Brazil. Their mothers most likely contracted Zika virus infection during the first trimester of pregnancy in early 2015. Six of the families lived in small towns far from the capital of Fortaleza, suggesting that Zika virus was widespread in Ceará at that time.

During November 2015–February 2016, the 7 autopsies were performed by the Service for Ascertaining Death (Ceará, Brazil). Real-time reverse transcription PCR (RT-PCR) of cerebrospinal fluid and tissue, performed at the Central Public Health Laboratory (Ceará), confirmed congenital Zika virus infection (*8*) (Table). We reviewed medical charts from live patients and their mothers; autopsy reports; and histopathologic reports from 4 pathologists who reviewed the hematoxylin and eosin–stained slides of brain, cerebellum, lung, heart, liver, spleen, kidney, and bladder. Samples were also tested for dengue virus by real-time RT-PCR and for dengue virus nonstructural protein 1 and IgM (*9*,*10*) (Table). After consent was obtained from families, the autopsies were performed as routine cause-of-death investigations. 

**Table:** Results of tests for Zika virus in CSF and organs and for dengue virus in brain and CSF for 7 neonates who died of congenital Zika virus infection, Brazil*

Neonate no.	Zika virus rRT-PCR								DENV			
	CSF	Brain	Lung	Heart	Liver	Spleen	Kidney		Brain rRT-PCR	CSF		
										rRT-PCR	IgM†	NS1‡
1	P	P	P	P	N	N	P		ND	N	N	N
2	P	P	N	P	P	P	P		ND	ND	N	N
3	P	P	P	P	P	P	P		ND	ND	N	N
4	P	P	N	N	N	N	N		N	DENV-1	P	P
5	P	P	P	P	P	P	P		N	N	N	N
6	P	ND	ND	ND	ND	ND	ND		N	N	N	N
7	P	ND	ND	ND	ND	ND	ND		ND	N	N	N


*CSF, cerebrospinal fluid; DENV, dengue virus; N, negative; ND, not done; NS1, nonstructural protein 1 (dengue virus antigen test); P, positive. †By antigen capture assay. ‡By ELISA.

Of the 7 mothers, all were HIV negative and 5 had had symptoms compatible with viral infection during the first trimester of pregnancy. Neonate survival times ranged from 30 minutes to 6 days after birth; 5 survived <1 hour, 1 died 48 hours after birth, and 1 survived for 6 days. Four neonates were male; for 1 of these in whom the genitalia were ambiguous (neonate 2), the sex was ascertained by identification of undescended testes at autopsy. Gestational ages ranged from 30 to 42 weeks (median 37 weeks) (Technical Appendix).

**Figure 1:**
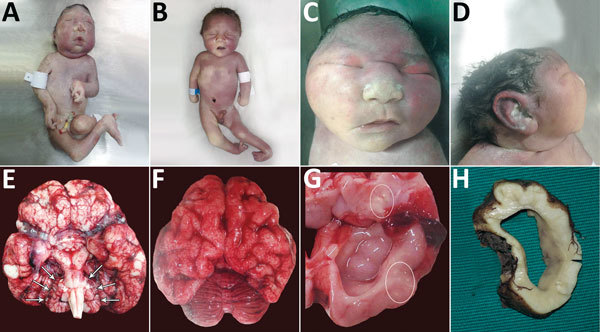
Physical signs in 4 of 7 neonates who died of congenital Zika virus infection, Brazil. A) Neonate 1: typical microcephaly phenotype; arthrogryposis in upper and lower limbs. B) Neonate 7: microcephaly without the typical microcephaly phenotype; arthrogryposis is also present. C) Neonate 3: typical microcephaly phenotype, with head circumference within reference limits, frontal view. D) Neonate 3: typical microcephaly phenotype, with head circumference within reference limits, profile view. E) Brain of neonate 2: symmetric cerebellar hypoplasia (arrows) and vascular congestion. F) Brain of neonate 3: pachygyria and severe vascular congestion. G) Brain of neonate 3: ventriculomegaly and macroscopic calcifications (circles). H) Brain of neonate 7: cross-section showing ventriculomegaly.

Brain weight was decreased in all neonates; however, body weight was within reference range for gestational age in all except neonate 6 (online Technical Appendix). For 6 neonates, microcephaly was present (Figure 1, panels A, B). One neonate for whom head circumference was within reference limits had morphologic changes typical of microcephaly (Figure 1, panels C, D). Cerebellar hypoplasia was present in neonates 1 and 2 (Figure 1, panel E) and pachygyria in neonate 3 (Figure 1, panel F). Ventriculomegaly was present in all neonates (Figure 1, panels G, H) and arthrogryposis in 6 (Figure 1, panels A, B).

**Figure 2:**
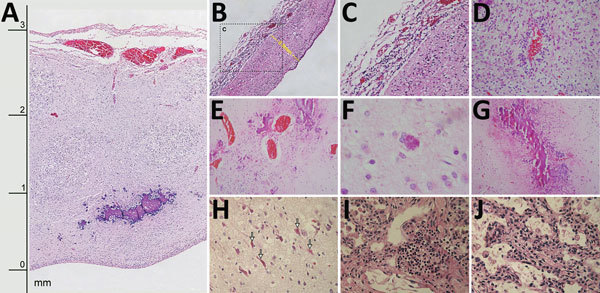
Histologic slides of tissues from 4 of 7 neonates who died of congenital Zika virus infection, Brazil. A) Neonate 1: severe cortical thinning (3 mm) with subventricular dystrophic calcification, reactive gliosis, and marked leptomeningeal congestion as well as marked depletion of neuronal precursors (original magnification ×10). B) Neonate 1: severe thinning of brain parenchyma (0.8 mm) with striking depletion of neuronal precursors (original magnification ×10). C) Neonate 1: lymphocytic leptomeningitis (enlargement of box in panel B; original magnification ×20). D) Neonate 6: white matter with lymphocytic perivascular cuffing and severe gliosis (original magnification ×40). E) Neonate 3: marked parenchymal vascular congestion and scattered coarse dystrophic calcification (original magnification ×20). F) Neonate 3: finely granular intracellular calcification (original magnification ×40). G) Neonate 7: band-like pattern of coarse dystrophic calcification at the junction of gray and white matter (original magnification ×10). H) Neonate 6: red neurons (arrows) in brain parenchyma (original magnification ×40). I) Neonate 1: focal interstitial lymphocytic pulmonary infiltrate (original magnification ×40). J) Neonate 1: expansion of alveolar septa with scattered lymphocytic and macrophage infiltrate (original magnification ×40).

All infants had thinning of the brain parenchyma with severe depletion of neuronal precursors (Figure 2, panel A). In some areas of the brain, the distance from the meningeal membranes to the ependymal epithelium was 3.0 mm (Figure 2, panel A); in 1 neonate, it measured only 0.8 mm (Figure 2, panel B).

Lymphocytic inflammation was observed in the meninges of 5 neonates (Figure 2, panels B, C) and in the brain parenchyma of 5; for 3 neonates, inflammatory processes were found in both areas. Areas of inflammation varied in intensity and distribution, but periventricular and perivascular cuffing were common (Figure 2, panel D). There was moderate to severe vascular congestion in 6 neonates (Figure 1, panels E, F; Figure 2, panel E). Macroscopic calcification was common (Figure 1, panel G). Three patterns of dystrophic calcification were identified microscopically: individual neuronal mineralization, a fine granular pattern (Figure 2, panel F), and a more coarse dystrophic pattern (Figure 2, panels A, E, G). Coarse calcification in a band-like form (Figure 2, panels A, G) was seen mainly at the junction of gray and white matter and in periventricular areas in association with the inflammatory process. Dystrophic calcification with neuronal mineralization was seen in all neonates. A variable degree of gliosis was present in all neonates, affecting predominantly white matter. Red neurons, neurons with increased cytoplasmic eosinophilia (reflecting acute neuronal injury), were seen in 6 neonates (Figure 2, panel H), and apoptosis was seen in 5 (online Technical Appendix). Additional findings included foci of brain hemorrhage, mainly in periventricular areas.

Pulmonary hypoplasia was present in all neonates (online Technical Appendix); relative lung weight (lung weight/body weight) was 0.004–0.01 g (*11*). Intra-alveolar hemorrhage was seen in 3 neonates; bleeding was severe in neonates 5 and 7. Interstitial lymphocytic pulmonary infiltration (Figure 2, panel I) and expansion of alveolar septa (Figure 2, panel J) was present in neonate 1, who died soon after birth. Fresh frozen lung tissue from that neonate was positive for Zika virus by RT-PCR (Table).

Liver specimens were available from 6 neonates; moderate to severe hydropic degeneration was found in 5 of these specimens. Round eosinophilic cytoplasmic structures suggestive of megamitochondria were observed in 2 neonates, mild to moderate steatosis and Councilman bodies (apoptosis) in 3, hepatocyte necrosis in 1 (neonate 7) (online Technical Appendix), and mild periportal lymphocytic inflammation in 1 (neonate 2).

Kidneys were available from 6 of the neonates. Focal glomerular sclerosis was present in neonates 2 and 6. In neonate 2, tissue was positive for Zika virus by RT-PCR (Table). Moderate lymphocytic cystitis was present in tissue from 1 available bladder. No histologic abnormalities were found on any of the hearts or spleens. Placental tissues were available from 4 neonates; common findings were fibrinoid necrosis, chorangiosis, and amnion hyperplasia. Dengue virus 1 was detected by RT-PCR in cerebrospinal fluid from neonate 4 (Table).

## Conclusions

Of the 7 Zika virus–infected neonates examined, 6 did not show intrauterine growth restriction, but all 7 had remarkably decreased brain weight (online Technical Appendix), emphasizing the neurotropism of Zika virus. These findings are similar to those earlier reported for congenital Zika virus infection (*3*–*7*). In contrast, an animal model of congenital Zika virus infection with similar neuropathologic damage was associated with striking global growth retardation (*12*).

The constellation of neuropathologic features (ventriculomegaly, mineralized neurons, and dystrophic calcification with band-like subcortical distribution) differs from features seen in other common infections associated with congenital abnormalities (e.g., TORCH [toxoplasmosis, other viruses, rubella, cytomegalovirus and herpesvirus infections]) and should raise suspicion for congenital Zika virus infection, warranting further workup. For instance, the pattern of calcifications seen in tissue from patients with congenital cytomegalovirus infection and in toxoplasmosis are predominantly periventricular (*13*).

Pulmonary hypoplasia, defined as lung weight:body weight ratio of <0.012 (1.2%), seemed to be a major factor determining death during the perinatal period (*11*). The occurrence of pulmonary hypoplasia and arthrogryposis was most likely a part of fetal akinesia deformation sequence, resulting primarily from central nervous system damage (*14*).

In addition, variable liver damage, a finding commonly seen with infection by other flaviviruses, was found in these neonates (*15*). The detection of Zika virus in tissues that did not show pathologic abnormalities could be the result of viremia or of the tissues sampled; the pathologic abnormalities were mostly focal (samples for PCR could have differed from those used for histopathologic examination). Another possibility could be cross-contamination during the sample collection.

 Our report confirms not only the neurotropism of the virus but also the occurrence of pathologic changes consistent with viral infection in multiple organs: liver (Councilman bodies and periportal lymphocytic infiltration), lungs (interstitial lymphocytic pneumonitis), and bladder (lymphocytic cystitis). PCR detection of Zika virus in liver, lung, and kidney tissue also strengthened our hypothesis that Zika virus can infect multiple tissues.

Technical Appendix Postmortem findings for 7 neonates with congenital Zika virus infection.
