# Supplementary material for: Postmortem Findings for 7 Neonates with Congenital Zika Virus Infection
Source: Emerg Infect Dis. 2017 Jul;23(7):1164–7. doi: 10.3201/eid2307.162019 (PMC5512501; doi:10.3201/eid2307.162019)
Supplement: Technical Appendix — Postmortem findings for 7 neonates with congenital Zika virus infection. [file 16-2019-Techapp-s1.pdf]

Article DOI: https://doi.org/10.3201/eid2307.162019

Postmortem Findings for 7 Neonates with Congenital Zika Virus Infection

Technical Appendix

Technical Appendix Table. Most important postmortem findings for 7 neonates with congenital Zika vírus infection*

GENERAL DATA						BRAIN						LUNG			LIVER		
Case	Gestational age, w/sex	Bodyweight (grams) / (RM±SD)	External findings	Head circum (cm)	Brain weight (grams) / (RM±SD)	Ventriculomegaly	Inflammation	Calcification	Gliosis	Red neuron (acute neuronal injury)	Congestion	Pulmonary hypoplasia	Interstitial pneumonitis	Hemorrhage	Hydropic degeneration	Steatosis	Apoptosis
1	30/F	950 (1,211 ± 330)	Microcephaly, arthrogryposis	23	25 (173 ± 30)	Yes	M, B	Band, PV, BG, BS	WM	BG	mod	Yes	mild	No	No	No	No
2	36/M	1,976 (2,246 ± 511)	Microceph. Arthrogryp	31	155 (292 ± 42)	Yes	M	WM	C, WM, CB	No	severe	Yes*	No	mod.	mod.	No	No
3	37/M	3,310 (2,424 ± 535)	Microceph. Arthrogryp	32	90 (319 ± 44)	Yes	M	C, WM, PV	WM	C	severe	Yes*	No	No	NA	NA	NA
4	40/F	2,460 (2,942 ± 603)	Microceph. Arthrogryp	26	90 (368 ± 51)	Yes	M, B	Band	WM, CB	C, BS	mod.	Yes	No	No	severe	No	No
5	40/M	3,330 (2,942 ± 603)	Microceph.	30	130	Yes	B	C, WM	WM	C	mod.	Yes	No	severe	severe	mod.	mod.
6	42/F	1,750 (3,267 ± 641)	Microceph. Arthrogryp	25	25 (395 ± 55)	Yes	M, B	PV	WM	C	mod.	Yes	No	No	mod.	mild	mod.
7	35/M	2,115 (2,040 ± 487)	Microceph Arthrogryp	31	160	Yes	B	Band, BG	WM	BS	No	Yes*	No	severe	mod.	mod.	mod.

*Circum, circumference. BG = basal ganglia, BS = brainstem, C = cortex, CB = cerebellum, M = meninges, B = brain parenchyma, NA = not available, PV = periventricular, WM = white matter RM = reference mean SD = standard deviation (*) = Per autopsy report.
